# Spotlight on systems vaccinology: a novel approach to elucidate correlates of protection

**DOI:** 10.1038/s41435-023-00247-2

**Published:** 2023-12-26

**Authors:** Henderson Zhu, Irina Chelysheva, Andrew J. Pollard, Daniel O’Connor

**Affiliations:** 1 Oxford Vaccine Group, Department of Paediatrics, University of Oxford, Oxford, UK; 2 NIHR Oxford Biomedical Research Centre, Oxford, UK

**Keywords:** Bacterial infection, Conjugate vaccines

**Fig. 1:**
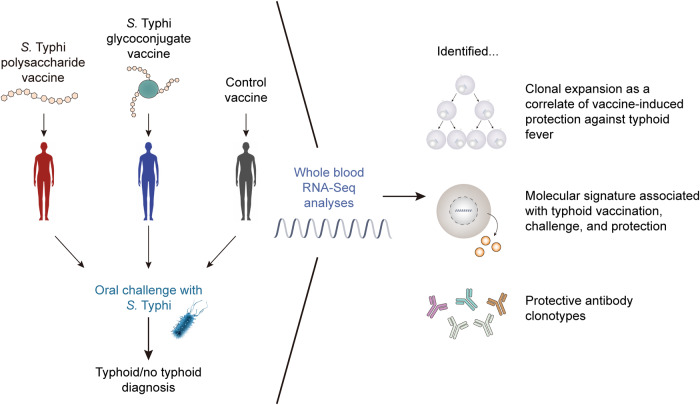
Schematic of the vaccine study and the outputs from the systems vaccinology pipeline.

Systems vaccinology employs multi-omic approaches, including transcriptomics, immunomics, proteomics, and metabolomics to detail the complex alterations in the biological systems of vaccine recipients after vaccination [1, 2]. Within this broad field, our recent article highlights a focused approach [3]. We describe a contemporary workflow, specifically using transcriptomic and immunomic analyses, tDE@Spr!ng19o establish correlates of vaccine-induced protection against typhoid fever (Fig. 1). Typhoid fever, caused by *Salmonella enterica* serovar Typhi (*S*. Typhi), continues to pose a substantial public health burden in many resource-limited regions of the world, resulting in approximately 100,000 deaths every year [4].

The substantial disease burden coupled with the emergence of antimicrobial resistant typhoid strains highlights the urgent need for effective vaccines to be deployed. Previous vaccines against typhoid fever failed to induce sufficient levels of protection in infants and toddlers. Therefore, there was a pressing need for alternative vaccines for paediatric immunisation. The fact that typhoid fever exclusively affects humans means that there are no non-human animal models for elucidating important features of the immune response to infection (e.g., correlates of protection). However, progress in this field has been accelerated by work of investigators at Oxford University, led by Professor Sir Andrew Pollard, establishing a controlled human infection model (CHIM), which quantified and contrasted vaccine efficacies of a licenced plain polysaccharide and a newer glycoconjugate typhoid vaccine [5].

A pivotal aspect of the current study was the generation of bulk RNA-sequencing data from blood samples collected at defined time points post-vaccination and post-oral typhoid challenge within the CHIM setting. As glycoconjugate vaccines and plain polysaccharide vaccines elicit protection through humoral immunity, coupling transcriptomic analyses with B-cell oriented immunomic analyses provided valuable insights. Transcriptomic analysis revealed that by 7 days post vaccination, recipients of both vaccines exhibited upregulation of humoral immunity-related genes such as *IGHG1* and *IGHV3-23*. These elevated signatures indicate a robust B-cell clonal expansion at 7 days post vaccination. Thus, quantifying the relative magnitudes of these clonal expansions may provide insight into protection. In agreement with previous findings, most glycoconjugate vaccine recipients who did not develop typhoid fever showed considerably higher levels of clonal expansion when compared with those who developed the disease. This observation enabled a correlation between the level of clonal expansion and protection to be established.

To identify the exact B-cell receptor clonotypes contributing to the clonal expansions seen after vaccination, we developed a high-throughput B-cell receptor (BCR) clonotype clustering method. This enabled the identification of an upregulated BCR cluster commonly seen amongst participants that did not develop typhoid fever post-challenge. This cluster’s BCR clonotypes shared sequence homology with antibodies experimentally determined to bind the Vi-capsular polysaccharide of *S*. Typhi. Thus, both computational and experimental evidence point to the protective role of these BCR clonotypes which matured following administration of both the plain polysaccharide vaccine and the glycoconjugate vaccine. This finding confirms a convergent humoral response while also identifying the protective component of the observed humoral response.

We think that immunomic analyses can serve as a complementary approach to other types of transcriptomic analyses for establishing correlates of protection, by elucidating protective B- or T-cell receptor clonotypes. In our study, we demonstrated that the B-cell clonal expansion can be effectively captured by transcriptomic analysis of blood samples taken post-vaccination, revealing novel correlates of vaccine-induced protection.
